# Pembrolizumab-induced hypothyroidism caused reversible increased serum creatinine levels: a case report

**DOI:** 10.1186/s12882-020-01775-z

**Published:** 2020-03-31

**Authors:** Natsumi Matsuoka, Kenji Tsuji, Eiki Ichihara, Takayuki Hara, Kazuhiko Fukushima, Kishio Toma, Shinji Kitamura, Kenichi Inagaki, Hitoshi Sugiyama, Jun Wada

**Affiliations:** 1 Department of Nephrology, Rheumatology, Endocrinology and Metabolism, Okayama University Graduate School of Medicine, Dentistry, and Pharmaceutical Sciences, 2-5-1 Shikata-cho, Okayama, 700-8558 Japan; 2 Department of Hematology, Oncology, Allergy and Respiratory Medicine, Okayama University Graduate School of Medicine, Dentistry, and Pharmaceutical Sciences, Okayama, Japan; 3 Department of Human Resource Development of Dialysis Therapy for Kidney Disease, Okayama University Graduate School of Medicine, Dentistry, and Pharmaceutical Sciences, Okayama, Japan

**Keywords:** Pembrolizumab, Hypothyroidism, Creatinine, Cystatin C

## Abstract

**Background:**

The advent of immune checkpoint inhibitors (ICIs) has significantly improved the prognosis of patients with advanced malignancies. On the other hand, these drugs might cause immune-related adverse events (irAEs) including endocrinopathies and nephropathies. Thyroid dysfunction is one of the most common irAEs. For ICIs-induced nephropathies, most cases are due to tubulointerstitial nephritis, which might require steroid treatment. Here, we report a patient with non-small cell lung cancer treated with ICI who developed increased serum creatinine (s-Cr) levels due to ICIs-induced hypothyroidism.

**Case presentation:**

A 57-year-old Asian man with refractory non-small cell lung cancer under ICIs therapy (pembrolizumab, an anti-programmed cell death-1 monoclonal antibody) developed increased s-Cr levels 5 months after the pembrolizumab initiation. His laboratory data, renal biopsy, and Gallium-67 scintigraphy findings denied pembrolizumab-induced tubulointerstitial nephritis. His renal function was correlated with thyroid function. Despite the increase of s-Cr levels, serum cystatin C levels were normal, which could be explained by the hypothyroidism. Levothyroxine treatment improved renal function as well as thyroid function. Then pembrolizumab was resumed, and both his thyroid and renal function remained normal level. Ultimately, we concluded that the increased s-Cr levels were caused by pembrolizumab-induced hypothyroidism.

**Conclusion:**

All clinicians involved in ICI treatment need to recognize the possible increase in s-Cr levels caused by ICIs-induced hypothyroidism, and we propose monitoring serum cystatin C levels to differentiate ICIs-induced hypothyroidism from tubulointerstitial nephritis before invasive renal biopsies or steroid treatment, which are recommended by the prescribing information for pembrolizumab, are performed.

## Background

Immune checkpoint inhibitors (ICIs) have been developed, and the advent of ICIs significantly improved the prognosis of patients with advanced malignancies. However, these drugs have been reported to lead to immune-related adverse events (irAEs), including endocrinopathies and nephropathies. Renal dysfunction occurs in 0.3% of the patients under the treatment of pembrolizumab, an anti-programmed cell death-1 monoclonal antibody [1], and most reported cases are due to tubulointerstitial nephritis [2]. Here we describe a case of reversible increased serum creatinine (s-Cr) levels due to hypothyroidism caused by pembrolizumab.

## Case presentation

**Fig. 1:**
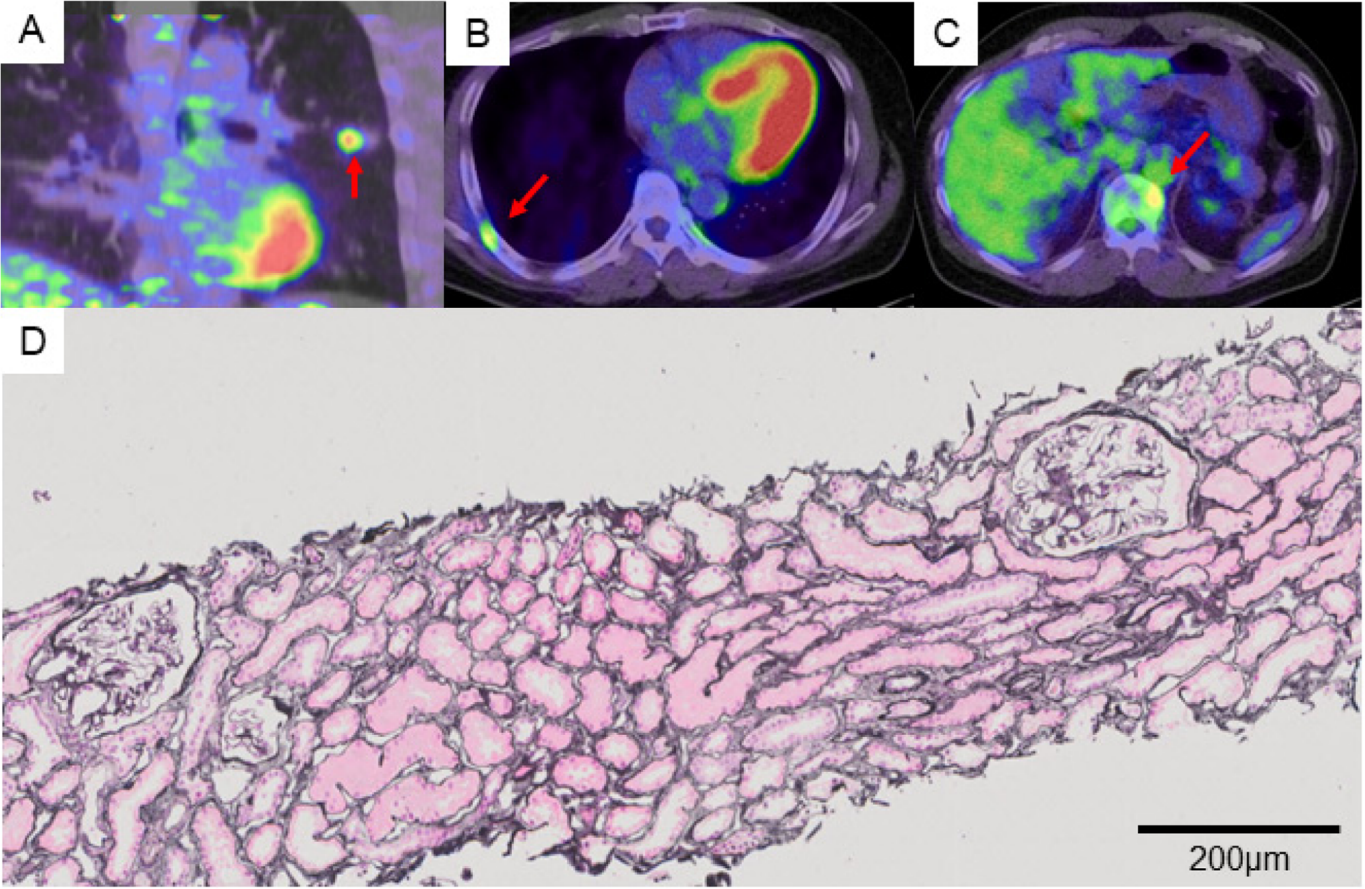
Imaging findings of lung CT and pathological findings of renal biopsy. **a** Positron emission tomography (PET)-CT picture showing a 2.5 cm-sized nodule on left upper lobe (arrow). **b**-**c** PET-CT pictures showing bone metastases on his right 7th rib and 11th thoracic vertebrae (arrows). **d** Light microscopy picture showing normal glomerular morphology without any evidences of tubulointerstitial abnormalities (periodic acid-methenamine-silver staining, × 40)

**Fig. 2:**
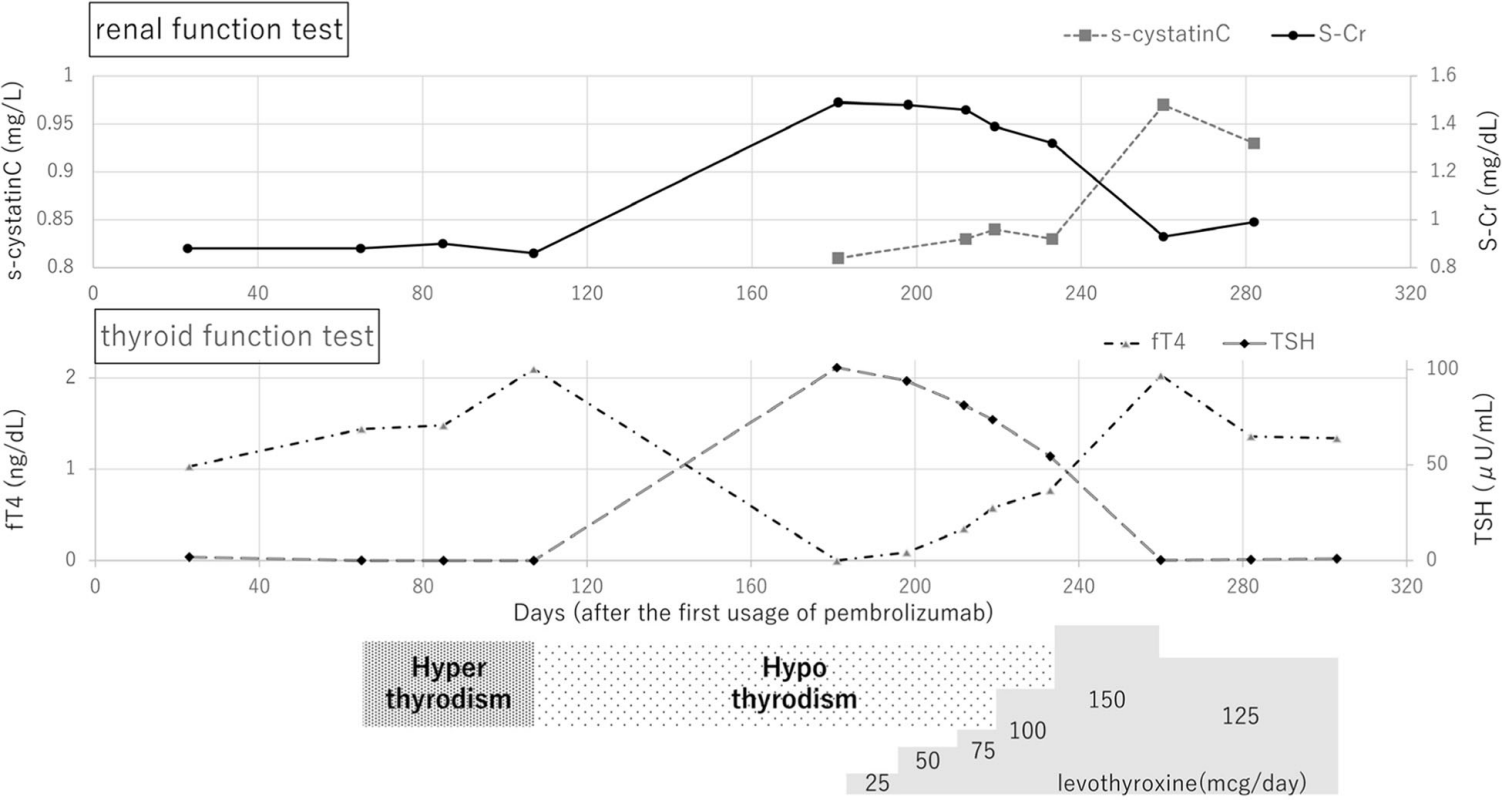
Clinical course. Renal and thyroid function tests after the first usage of pembrolizumab

A 57-year-old Asian man was diagnosed as lung adenocarcinoma with several bone metastasis (non-small cell lung cancer (NSCLC), T1cN0M1c stage IVB, epidermal growth factor receptor (EGFR): negative, anaplastic lymphoma kinase (ALK): negative, programmed death ligand 1 (PDL1) tumor proportion score: 5%, Fig. 1a-c), and he was initially treated with cisplatin and pemetrexed for 4 months, followed by 2nd line pembrolizumab treatment (200 mg/3 weeks). Although his lung tumor decreased in size, levels of s-Cr gradually increased (from 0.88 mg/dl to 1.49 mg/dl) and the estimated glomerular filtration rate (eGFR) decreased (from 69.9 ml/min/1.73m^2^ to 39.3 ml/min/1.73m^2^) within 5 months after the pembrolizumab initiation (Fig. 2). According to the Keytruda® prescribing information [3], pembrolizumab was discontinued, and he was admitted to our hospital. At admission, his height was 172.8 cm, his body weight was 88.1 kg, and his body mass index was 29.4 kg/m^2^. His body temperature was 36.4 °C, his pulse was 67 per minute, and his blood pressure was 144/92 mmHg. There were no episodes of hypotension at home or office visit. Electrocardiogram and computed tomography (CT) analyses denied arrhythmia, myocarditis or pericarditis, which could influence renal perfusion. There was no elevation in the C-reactive protein (CRP) levels. Despite the increase in s-Cr levels, serum cystatin C (s-cystatin C) levels were within the normal range (0.81 mg/L). 24-h creatinine clearance (Ccr) was 62.8 ml/min and urine creatinine was 1.8 g/day. Antinuclear and antineutrophil antibodies were negative. Urinalysis showed neither hematuria nor proteinuria nor an increase in urinary β_2_-microglobulin (β_2_MG) and N-acetyl-β-glucosaminidase (NAG) levels. Gallium-67 scintigraphy showed no uptake in the kidneys. A renal biopsy was performed, which revealed neither glomerular nor tubulointerstitial abnormalities (Fig. 1d), thus denying pembrolizumab-induced tubulointerstitial nephritis and glomerulonephritis.

Retrospective evaluations revealed that the patient had developed asymptomatic hyperthyroidism with anti-thyroglobulin antibodies (23 IU/mL) and anti-TSH receptor antibodies (TRAb) (6.31 U/l) 3 months after the initiation of pembrolizumab, which was followed by asymptomatic hypothyroidism without adrenal insufficiency (Fig. 2). In summary, we diagnosed the patient with pembrolizumab-induced painless thyroiditis. Since the increase in s-Cr levels paralleled the decline in free T4 (fT4) levels, we suspected an association between hypothyroidism and increased s-Cr levels. Levothyroxine at a dose of 25 μg daily was prescribed and gradually increased. Levothyroxine treatment improved thyroid function tests as well as s-Cr levels, while the levels of s-cystatin C were elevated. 24-h Ccr after the improvement of increased s-Cr levels (s-Cr: 1.00 mg/dl, eGFR: 61.5 ml/min/1.73m^2^) was 50.7 ml/min and urine creatinine was 1.11 g/day. Without any abnormalities on renal histology in renal biopsy or any signs of other causes of renal dysfunction, such as hypotension and cargiological abnormalities, we concluded that the increased s-Cr levels were most likely caused by the rapid decline of thyroid function during the transition from hyperthyroidism into hypothyroidism induced by pembrolizumab. Pembrolizumab was resumed, and the patient’s thyroid and renal function remained normal (Fig. 2).

## Discussion and conclusions

This is the first case of reversible increased s-Cr levels caused by ICI-induced hypothyroidism. Pembrolizumab is a humanized monoclonal anti-PD1 antibody that induces an immune response against tumor tissues. It has been successful in inducing remission in patients with severe metastatic disease, who are often refractory to other chemotherapeutic agents. In refractory NSCLC, pembrolizumab prolongs overall survival and progression-free survival [3]. However, irAEs occur in 20% of patients on pembrolizumab [4]. One of the most frequent irAEs caused by pembrolizumab is thyroid disorders (hypothyroidism, 8%; hyperthyroidism, 6%) [4]. It was reported that nephritis occurred in 9 (0.3%) of 2799 patients receiving pembrolizumab, including grade 2 (0.1%), grade 3 (0.1%), and grade 4 (< 0.1%) nephritis [1]. In the majority of the cases, renal dysfunction under pembrolizumab treatment is due to tubulointerstitial nephritis [2]. Therefore, the cessation of pembrolizumab, renal biopsies, and steroid treatment (prednisolone 1–2 mg/kg/day) are recommended in cases of grade 2-4 renal failure in the Common Terminology Criteria for Adverse Events (CTCAE) [1]. However, in our case, careful evaluation, including renal biopsy, denied pembrolizumab-induced tubulointerstitial nephritis and revealed that pembrolizumab may have caused hypothyroidism-related increase in s-Cr levels, which was able to be resolved with levothyroxine treatment; thus, neither steroid therapies nor cessions of pembrolizumab were required.

There is accumulating evidence showing a strong correlation between changes in thyroid status and changes in renal function [5]. It has been reported that s-Cr levels decrease (− 17.6%) in hyperthyroidism compared with those seen with normal thyroid function, whereas an elevation in s-Cr levels is seen in hypothyroidism compared with that seen with normal thyroid function (+ 11%) [6]. In addition, a recent study indicated a significant decline in eGFR after patients with hyperthyroidism became euthyroid, suggesting that hyperthyroidism might mask mild renal failure [7]. It has also been reported that eGFR declines in 55% of adults with hypothyroidism, and thyroid hormone replacement usually resolves renal dysfunction [8, 9]. Although the detailed mechanisms by which hypothyroidism causes declines in eGFR are still unclear, it is suggested that hypothyroidism might decrease renal function in multiple mechanisms. These include decreased kidney mass [10], reduced renal perfusion pressure because of decreased cardiac output and increased vascular resistance [11–13], reduced sensitivity to the body’s sympathetic drive and renin-angiotensin-aldosterone system activity [14], and possible rhabdomyolysis [15]. In addition, it has also been reported that increased s-Cr levels under hypothyroidism might be associated with increased circulating Cr, suggesting hypothyroidism might increase s-Cr levels independent of GFR. These mechanisms include increased release of Cr from muscle tissue due to myopathy and rhabdomyolysis [16], and increased conversion from creatine into creatinine [17]. Indeed, in our case, re-evaluation of renal function revealed no increase in 24-h Ccr after the levothyroxine treatment despite the apparent improvement in s-Cr levels. In addition, a decrease in urine Cr after the levothyroxine treatment (from 1.8 g/day to 1.11 g/day) was observed. These observations suggest that increased s-Cr levels under hypothyroidism might be mainly due to the increased Cr supply rather than due to decreased GFR. Further study is required to uncover the exact mechanisms of increased s-Cr levels under hypothyroidism.

In contrast to the increase in s-Cr levels, the levels of s-cystatin C levels normally decrease under hypothyroidism [18]. It has been reported that s-cystatin C levels decline in hypothyroidism due to reduced production, consequent to reduced cellular metabolism [18], and this result is different from that seen with other causes of renal dysfunction. Indeed, in our case, s-cystatin C levels were within the normal range despite the increased s-Cr level on admission, and levothyroxine treatment reversed the increase in s-Cr levels while it elevated s-cystatin C levels. These findings are compatible with hypothyroidism-induced increase in s-Cr levels. Importantly, both tubulointerstitial nephritis and hypothyroidism-induced increase in s-Cr levels are unlikely to show apparent proteinuria or hematuria; Thus, these two different kinds of pathologies are difficult to differentiate by urinalysis. Therefore, monitoring both s-Cr and s-cystatin C levels might help us differentiate the causes of increased s-Cr levels.

Immune-related endocrine toxicities, including thyroid dysfunction, are usually irreversible. Interestingly, overall survival with pembrolizumab was significantly higher in subjects who developed thyroid dysfunction under pembrolizumab treatment than those who did not develop thyroid dysfunction (hazard ratio, 0.29; 95% confidence interval (CI) 0.09–0.94; *p* = 0.04) [8], suggesting these patients might not need to stop permbrolizumab treatment but rather could continue treatment with levothyroxine. Therefore, hypothyroidism during pembrolizumab treatment needs to be recognized quickly and to be treated appropriately so that patients can keep using pembrolizumab and prolong their overall survival. Considering the high frequency of pembrolizumab-induced hypothyroidism, our case is unlikely to be rare, and more similar cases could appear. The number of cases might be underestimated because some cases might be misdiagnosed as tubulointerstitial nephritis and subjected to steroid treatment. Since not so many clinicians are aware that hypothyroidism may cause increased s-Cr levels, it is urgent to highlight possible hypothyroidism-caused increase in s-Cr levels, which might be reversed with levothyroxine treatment. Nevertheless, it may take a relatively long time until s-Cr levels return to baseline with the levothyroxine treatment under hypothyroidism-induced increase in s-Cr levels. Indeed, it took approximately 2 months in our case. Since the delay of steroid treatment in tubulointerstitial nephritis could lead to irreversible renal damage, renal biopsy is still am important option to differentiate these causes when physicians are not confident in ruling out the possibility of tubulointerstitial nephritis.

In conclusion, all clinicians involved in ICI treatment need to recognize possible hypothyroidism-caused increase in s-Cr levels, which can be reversed with levothyroxine treatment. We propose monitoring s-cystatin C levels routinely as well as thyroid function under ICI treatment to differentiate hypothyroidism-related increase in s-Cr levels from tubulointerstitial nephritis before renal biopsies or steroid treatment are performed.

## Data Availability

All data generated or analysed during this study are included in this published article.

## Abbreviations

ICIs: Immune checkpoint inhibitors
irAEs: immune-related adverse events
NSCLC: Non-small cell lung cancer
EGFR: Epidermal growth factor receptor
ALK: Anaplastic lymphoma kinase
PDL1: Programmed death ligand 1
s-Cr: serum creatinine
eGFR: estimated glomerular filtration rate
CT: Computed tomography
CRP: C-reactive protein
s-cystatin C: serum cystatin C
Ccr: Creatinine clearance
β_2_MG: β_2_-microglobin
NAG: N-acetyl-β-glucosaminidase
TRAb: anti-TSH receptor antibody
fT4: free T4
CTCAE: Common Terminology Criteria for Adverse Events
PET: Positron emission tomography
CI: Confidence interval
